# Ultra-stable liquid crystal droplets coated by sustainable plant-based materials for optical sensing of chemical and biological analytes†

**DOI:** 10.1039/d3tc00598d

**Published:** 2023-04-11

**Authors:** Shikha Aery, Adele Parry, Andrea Araiza-Calahorra, Stephen D. Evans, Helen F. Gleeson, Abhijit Dan, Anwesha Sarkar

**Affiliations:** a Department of Chemistry and Centre for Advanced Studies in Chemistry, Panjab University Chandigarh 160014 India abhijit@pu.ac.in; b Food Colloids and Bioprocessing Group, School of Food Science and Nutrition, University of Leeds LS2 9JT UK A.Sarkar@leeds.ac.uk; c School of Physics and Astronomy, University of Leeds LS2 9JT UK; d Department of Applied Chemistry, Maulana Abul Kalam Azad University of Technology, Simhat Haringhata West Bengal 741249 India

## Abstract

Herein, we demonstrate for the first time the synthesis of ultra-stable, spherical, nematic liquid crystal (LC) droplets of narrow size polydispersity coated by sustainable, biodegradable, plant-based materials that trigger a typical bipolar-to-radial configurational transition in dynamic response to chemical and biological analytes. Specifically, a highly soluble polymer, potato protein (PoP) and a physically-crosslinked potato protein microgel (PoPM) of ∼100 nm in diameter, prepared from the PoP, a byproduct of the starch industry, were compared for their ability to coat LC droplets. Although both PoP and PoPM were capable of reducing the interfacial tension between water and *n*-tetradecane <30 mN m^−1^, PoPM-coated LC droplets showed better stability than the PoP-coated droplets *via* a Pickering-like mechanism. Strikingly, the Pickering LC droplets outperformed PoP-stabilized droplets in terms of dynamic response with 5× lower detection limit to model chemical analytes (sodium dodecyl sulphate, SDS) due to the difference in SDS-binding features between the protein and the microgel. To eliminate the effect of size polydispersity on the response, monodisperse Pickering LC droplets of diameter ∼16 μm were additionally obtained using microfluidics, which mirrored the response to chemical as well as biological analytes, *i.e.*, primary bile acid, an important biomarker of liver diseases. We demonstrate that these eco-friendly microgels used for creating monodisperse, ultra-stable, LC complex colloids are powerful templates for fabricating next generation, sustainable optical sensors for early diagnosis in clinical settings and other sensing applications.

## Abstract 2

The plant-based polymer and microgel act as effective emulsifiers for liquid crystal (LC)-in-water emulsions where microgels provide better storage stability and response to chemical and biological analytes with 5× lower detection limit than the protein.

## Graphical abstract

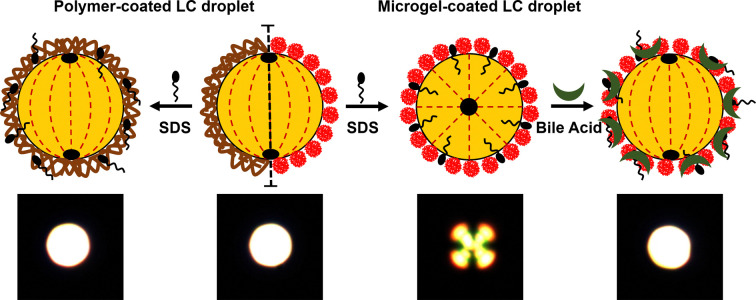

## 1. Introduction

Liquid Crystal (LC)-in-water emulsions are a fascinating class of anisotropic droplet-based optical sensors that consist of micrometer-sized LC droplets dispersed in a continuous aqueous phase for widespread optical, photonics and sensing applications.^1–4^ These functional materials behave as a sensitive communicator for a broad range of targeted species, such as endotoxins, DNA, viruses *etc.*, due to their stimuli-responsive director configurations.^5–10^ The soft LC droplets possess highly sensitive anisotropic optical properties (as LCs are birefringent materials) such that they exhibit distinct optical appearances depending on the internal ordering of LC molecules within the droplet. The targeted aqueous analytes upon dynamic adsorption at the LC–water interface may alter the local ordering of the interfacial LC molecules. The new orientation established at the interface upon adsorption of the analyte rapidly propagates into the bulk LC within the droplets and triggers the re-organization of the internal ordering of LC molecules inducing the director configurational transition within the droplets, which can be easily imaged using polarized light microscopy. Ultimately, this allows for the label-free detection of aqueous analytes and eliminates the need for complex and expensive detection systems for signal transduction. Due to their large surface area, high spatial resolution and sensitivity, tuneable optical properties and stimuli-responsive director configuration, LC droplets have attracted a great deal of attention for developing photonic devices, chemical and biological sensors.

For the quantification study of analytes, the number of LC droplets undergoing a transition as a function of analyte concentration is the measured entity. However, such quantification of configurational states of the LC droplets is hindered owing to the limited stability of a few hours, poor shelf life, polydispersity and sedimentation of LC droplets, thus resulting in unreliable data. To address this issue, extensive research efforts have been made that involve the modification of bare LC–water interface with the surfactants,^7^ polymers^11,12^ and particles.^13,14^ They provide varying degrees of electrostatic and steric stabilization to the LC droplets and hinder droplet coalescence and sedimentation. Further, the presence of such interfacial materials introduces new behaviours to the LC droplet assembly that are fundamentally different from the bare LC droplets. Additional interfacial tailoring approaches are reported in the literature, such as the incorporation of the LC droplets in polymer capsules^15^ or in polymer-based hydrogel films to improve their longer-term stability.^16^

Our group has established that the submicron-sized microgels that are intermediate between the hard spheres and the polymers also behave as effective stabilizers for LC-in-water emulsions.^13,14^ They spontaneously and irreversibly adsorb at the LC–aqueous interface by forming a monolayer or multilayer and provide Pickering-like stabilization to the LC droplets. They effectively enhance their shelf life without inhibiting the optical sensing behaviour. The interfacial pores on the LC droplet surface further act as effective nanoscopic windows to allow for the diffusion of small molecules, such as single-chain surfactants, through which they can enter and interact with the LC droplets allowing optical transitions. However, to date, such microgels for LC applications have been made with synthetic polymers (*e.g.*, poly(*N*-isopropylacrylamide) (*p*NIPAM)) requiring petrochemical-based solvents for synthesis^17^ or animal proteins (whey protein) with significantly high greenhouse gas emissions,^18^ which raises sustainability concerns. It is pertinent, therefore, to consider a greener fabrication route for the emulsifier used in LC droplet-based detection, with consideration given to the importance of biocompatibility, low carbon emissions and non-toxicity requirements whilst still retaining exceptional stability as well as a dynamic response on an analyte stimulation.

Recently, plant-based proteins (biopolymer) and lab-synthesized proteinaceous microgel particles have been determined to be effective, eco-friendly emulsifiers for fabricating conventional and Pickering food-grade emulsions, respectively.^19–21^ Being biocompatible, sustainable, cost-effective and non-toxic, they are in high demand to replace dairy proteins and particles. In particular, the potato protein (PoP) has attracted significant research attention as it is derived from the waste material generated during the industrial starch manufacturing process.^22^ Their soluble fraction mainly consists of patatins (∼40%), protease inhibitors (∼50%) and oxidative enzymes (∼10%). More importantly, unlike other plant proteins, this is a largely soluble protein allowing various technological applications.^23^ Although the extraction process of PoP leads to a significant loss of nutritional value, the foaming, emulsifying, and gelling abilities of the PoP has captured significant research interests.^24–26^ Liu *et al.* have prepared potato protein microgels (PoPM) by uncontrolled denaturation of the PoP and used them as Pickering stabilizers for corn oil-in-water emulsions.^27^ However, there is currently a lack of in-depth understanding on the stabilization and mechanism of dynamic response in the LC droplets when stabilized by plant-based materials. Whether the self-aggregating nature of PoP^28^ influences the interfacial properties at the LC–water interface remains unknown. Also, the question remains whether or not such plant-based materials, if adsorbed at the LC–water interface, interfere with the response properties by interacting with the analytes themselves.

Therefore, in this work, we firstly asked the question of which of the PoP polymer (classic emulsifier) or the microgel (Pickering emulsifier) can act as a better emulsifier for the LC-in-water emulsions-based optical sensors for longer-term kinetic stability. Secondly, is there any difference in the detection thresholds for analyte detection between the plant-based polymer and the microgel when used at the LC–water interface. Finally, we asked whether or not plant-based materials interfered with the detection of biological analytes such as bile acids (BAs), a biomarker for the early diagnosis of liver diseases (LDs).^29^ Using a comprehensive suite of complimentary techniques, such as interfacial tensiometry, rheology, circular dichroism, microscopy across scales, zeta-potential, and light scattering, we demonstrated the remarkable performance of using plant-based microgels at the interface. Such microgels provide not only ultra-stability but also a 5× lower detection threshold for analytes. The use of a single-step microfluidic procedure further confirmed the ability of these microgels to form monodisperse LC droplets capable of undergoing the analyte-triggered configurational transition. To our knowledge, this is the first report involving a comparative study on the synthesis and mechanism of the response of polymer *vs.* Pickering stabilizer using plant-based materials for LC droplets, paving the way forward for next generation sustainable LC-based biosensing applications.

## 2. Experimental

### 2.1 Experimental materials

Potato protein isolate with 90% protein content was purchased from Henley Bridge Ingredients Limited (Lewes, UK). The small nematic temperature window of the commonly used LC, such as nematic, 4-cyano-4′-pentylbiphenyl (5CB) (18–35 °C) restricts their use in clinical settings.^14^ Hence, a nematic LC mixture, E7, with a wide nematic range between −62 to +61 °C^30^ was purchased from Synthon Chemicals GmbH & Co. KG (Bitterfeld-Wolfen, Germany). E7 consists of four nematic cyanoparaphenylene LCs, *i.e.*, 4-cyano-4′-*n*-pentylbiphenyl (5CB) (51.0 wt%), 4-cyano-4′-*n*-heptylbiphenyl (7CB) (25.0 wt%), 4-(4′-*n*-octyloxyphenyl)benzonitrile (8OCB) (16.0 wt%), and 4-cyano-4′-*n*-pentyl-*p*-terphenyl (5CT) (8.0 wt%).^31^*N*-tetradecane was purchased from Alfa Aesar (Massachusetts, US). Sodium azide, fast green, bovine serum albumin (BSA), sodium cholate hydrate (NaCh) (3α,7α,12α-trihydroxy-5β-cholan-24-oic acid sodium salt), sodium dodecyl sulfate (SDS), bradford reagent, and the phospholipids, 1,2-dioleoyl-*sn*-glycero-3-phosphocholine (DOPC) and 1,2-dioleoyl-*sn*-glycero-3-phospho-(1′-rac-glycerol) (sodium salt) (DOPG) were purchased from Sigma-Aldrich (Dorset, UK). Glass syringes and polytetrafluoroethylene (PTFE) tubing for microfluidic droplet production were purchased from Scientific Glass Engineering (Ringwood, UK) and Bohlender (Grunsfeld, Germany), respectively. 23 gauge luer lock syringe tips were purchased from Metcal (California, US). Clear microscopy slides (0.8–1.0 mm thick) and 1.5 mL centrifuge tubes were purchased from Fisher Scientific (Pittsburgh, US). Analytical grade reagents were used unless otherwise specified. Milli Q water with a resistivity of 18.2 MΩ cm at 25 °C was obtained from the Milli Q apparatus from Millipore Corp. (Bedford, US) and was used as a solvent in all experiments. The secure seal spacers (9 mm diameter and 0.12 mm depth) and cover slips (12 mm) were obtained from Invitrogen, Thermo Fisher Scientific (Oregon, US) and VWR (Darmstadt, Germany), respectively.

### 2.2 Preparation of potato protein (PoP), potato protein microgels (PoPM) and LC-in-water emulsions

The aqueous solutions of PoP and aqueous dispersions of PoPM were prepared from potato protein isolate using the method reported in the ESI.† The microgels were produced using a top–down shearing approach of thermally crosslinked PoP gel used previously for other protein types elsewhere.^32^ The E7 LC-in-water emulsions were prepared using two different techniques: High pressure homogenization and Microfluidics, in the presence of polymer PoP, particle PoPM and phospholipid DOPC/DOPG as emulsifiers. The LC emulsions without additional emulsifiers were used as control. The detailed protocol of emulsions preparation is provided in the ESI.†

### 2.3 Characterization of the PoP solution, PoPM dispersion and LC-in-water emulsions

The prepared PoP and PoPM were characterized to obtain information on their size distribution, hydrodynamic diameter, *d*_H_, and polydispersity index (PDI) in aqueous dispersion using dynamic light scattering (DLS), differences in the conformation (secondary and tertiary structure) by circular dichroism (CD) spectroscopy, interfacial properties by measuring the interfacial tension at *n*-tetradecane–water interface in the presence of PoP and PoPM using Pendant drop method. Further, information related to the viscoelastic behaviour of PoP and average mesh size, *ξ*,^33^ of PoPM was obtained *via* a rheology study.

Furthermore, the prepared LC emulsions were characterized using confocal laser scanning microscopy (CLSM) and cryogenic-scanning electron microscopy (cryo-SEM) to investigate the location and morphology of PoP and PoPM at the LC–water interface, respectively. The size distribution and polydispersity of E7 droplets was investigated in terms of Sauter mean diameter, *D*_[3,2]_, De Broucker Diameter, *D*_[4,3]_, and PDI, respectively. The adsorption efficiency, *α*, and effective adsorption density, *Γ*_ads_, of PoP and PoPM were determined. Lastly, the surface charge of PoP, PoPM and the emulsions was reported in terms of *ζ*-potential using Zetasizer. The protocol followed for all characterization techniques is reported in detail in the ESI.†

### 2.4 Response study of the emulsions

Here, the response of PoP and/or PoPM-stabilized E7 emulsions to a model chemical analyte, *i.e.*, SDS and a biological analyte, *i.e.*, NaCh, was studied by imaging the internal configuration of E7 droplets using Leica DM 2700 P microscope, equipped with the cross polarizers operating in transmission mode with 50× objective and DeltaPix Invenio 3SII camera. Before the response study, the emulsions were washed to remove the unadsorbed emulsifier, which can interfere in the response measurements. For this, the emulsions were centrifuged at 1000 rpm for 3 min three times and re-dispersed in Milli Q water.

To study the response of emulsions to analytes, 300 μL of emulsion prepared either by homogenization or by the microfluidic route were mixed with aqueous solutions of different concentrations of analytes, and incubated for 30 min with occasional gentle shaking to allow optimum adsorption of analytes at E7–water interface. The response study for NaCh involved a two-step process. Firstly, the emulsions were incubated with SDS and the SDS-emulsion mixture was subsequently exposed to NaCh. 100 polarized optical micrograph (POM) or bright field microscopy images of each sample were recorded and analysed with ImageJ software (version 1.48r, National Institute of Health, Bethesda, USA).

## 3. Results and discussion

**Fig. 1:**
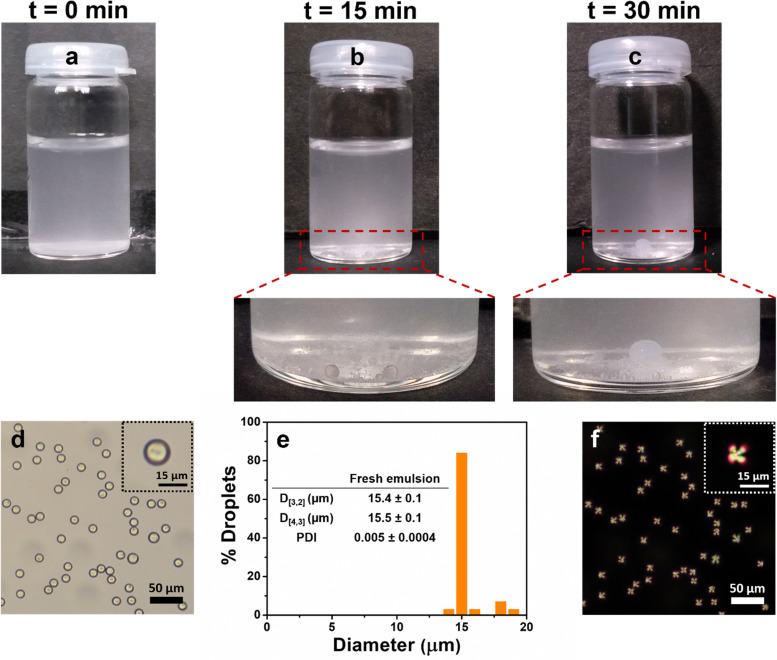
Visual images of bare E7-in-water emulsions immediately after preparation (a), after storage for 15 min (b) and 30 min (c), respectively. Bright field microscopy image (d) and droplet size distribution (e) of the freshly prepared phospholipid-coated E7-in-water emulsions with inset table showing the mean size parameters. Polarized optical microscopy image (f) of freshly prepared 1 : 1 DOPC : DOPG phospholipid-coated E7 droplets. Data in (e) represent average of three independent readings on triplicate samples (*n* = 3 × 3).

In order to understand the challenge in the stability of LC droplets as well as their response to analytes, we first synthesized bare and phospholipid-stabilized emulsions to serve as controls. Bare E7-in-water emulsions obtained from high pressure homogenization of E7 LC and water were found to be very unstable with low shelf life wherein the LC droplets lasted for about 15 min and then separated out from the emulsions (Fig. 1a–c). The large interfacial area of uncoated E7 droplets resulted in their coarsening such that visual sedimentation of LC was observed after just 30 min of preparation. The preparation of bare E7 droplets using microfluidics was unsuccessful, with no droplets being obtained in the exit channel.

In another attempt, E7 droplets in the presence of amphiphilic phospholipids (1 : 1 DOPC : DOPG phospholipid, the total concentration of 5 mg mL^−1^) were fabricated using a microfluidic device, which resulted in phospholipid-coated E7 droplets as shown by the bright field image (Fig. 1d). The droplets were monodisperse with mean diameters, *D*_[3,2]_ and *D*_[4,3]_ of 15.4 and 15.5 μm, respectively and very low PDI of 0.005 (Fig. 1e) in line with previous reports.^31^ However, the adsorbed phospholipid interacted with the surface LC molecules to induce perpendicular (homeotropic) ordering, as evident from a typical Maltese cross seen for the so-called pre-radial configuration^34^ of the resultant phospholipid-coated E7 LC droplets (Fig. 1f). More importantly, the sample was stable for a few hours to days. It has already been shown that such droplets can be used for the detection of analytes,^31^ but their poor stability is a serious problem in real devices. This confirms the need for stable LC droplets that clearly exhibit a sharp transition from a non-radial alignment upon stabilization to a radial alignment upon response to analytes for clear detection and quantitation.

Finally, in order to obtain stable E7-in-water emulsions with longer shelf life using an emulsifier that itself will not alter the LC configuration and have optical sensing behavior, two new plant-based materials, a polymer (PoP) and a microgel (PoPM), were tested (see Experimental section in ESI,† for fabrication).

### 3.1 Comparison of structural and interfacial characteristics of the plant-based polymer *vs.* microgels

**Fig. 2:**
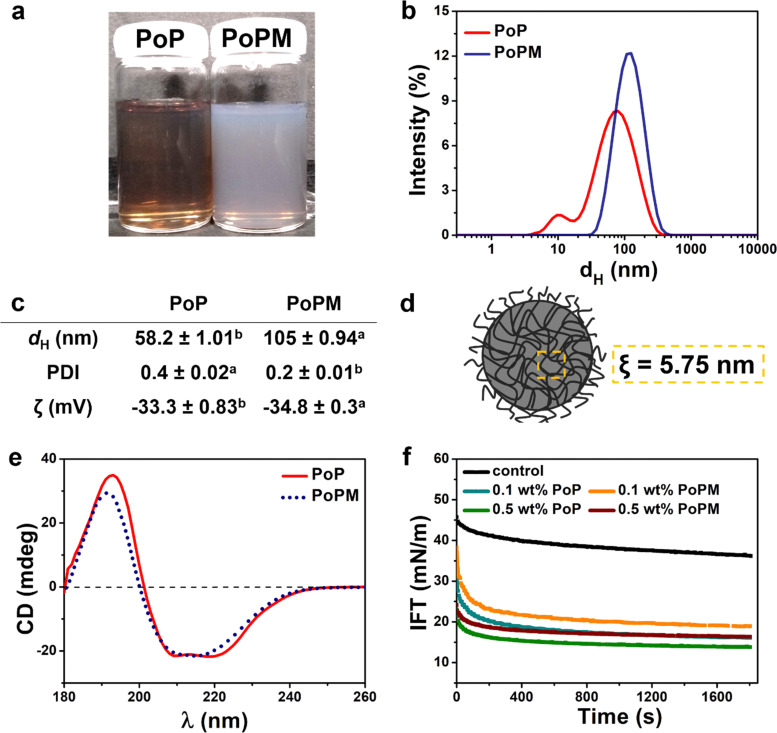
Visual images (a), particle size distribution obtained by dynamic light scattering (b), physicochemical properties (c) of PoP solution and an aqueous dispersion of PoPM (each containing 5.0 wt% potato protein isolate) with a schematic illustration of PoPM (d) showing the mesh size (*ξ*) obtained using rheology measurement of PoP parent gel. Far UV CD spectra of PoP and PoPM dispersions (e) and their interfacial tension measurements (IFT) (f) as a function of concentration at the *n*-tetradecane-water interface. Control in (f) represents the bare *n*-tetradecane-water interface without any added protein or microgels. Data in (b), (c), (e) and (f) represent the average of three independent readings on triplicate samples (*n* = 3 × 3). Samples in the same row with different superscripts differ significantly (*p* < 0.05) according to Tukey's test (c).

The formation of microgels was visually evident in Fig. 2a, where the protein transformed from a clear transparent liquid (PoP) to a turbid, milky appearance in PoPM. Generally, plant protein monomers range in size of few nanometers as previously reported in X-ray scattering and atomic force microscopy.^35,36^ Here, DLS revealed that the PoP had a bimodal, broad size distribution with two peaks appearing at 10 and 80 nm (Fig. 2b), owing to the inherently aggregating nature of storage proteins.^23^ As one might expect, PoPM had a larger size as compared to PoP, but strikingly, the former exhibited a monomodal and narrow size distribution with a single peak at around 125 nm. This implies that the thermal crosslinking of the PoP hydrogel and controlled shearing of PoP-gel *via* top-down fabrication approach resulted in a monodisperse PoPM dispersion with *d*_H_ ∼ 105 nm and PDI as low as 0.2 (Fig. 2c). Both PoP and PoPM were negatively charged with average *ζ*-potential values of −33.3 and −34.8 mV, respectively at pH 6.15 highlighting that the charged groups were still located at the surface for the microgels (Fig. 2c). With the isoelectric point (pI) ranging in between pH 4.5 and 5.0,^37^ the electrostatic repulsion between the negatively-charged surface provided a higher colloidal stability to both PoP and PoPM dispersions against macroscopic sedimentation over at least six months of storage.

It is noteworthy, that in a LC droplet, analytes may not only diffuse *via* the interfacial pores, *i.e.*, the spaces where the droplet is not covered by an emulsifier, but also through the meshes of a microgel, particularly in the case of PoPM. In order to calculate the mesh size (*ξ*) of these sub-micron-sized PoPM, the viscoelasticity of the 10.0 wt% PoP-heat set parent gel was measured with the assumption that the hydrogel and the microgel had the same viscoelasticity (see the temperature and frequency sweep of the gels Fig. S1a and b, ESI†). The weak dependence of both storage (*G*′) and loss moduluii (*G*′′) on angular frequency (*ω*), absence of crossover points between *G*′ and *G*′′ and predominance of *G*′ over *G*′′ implied that the patent PoP-gel and, henceforth, the PoPM exhibited strong gel-like behavior, *i.e.*, characteristic of typical viscoelastic solids.^33^

The average *ξ* of PoP-gel and/or PoPM was calculated to be 5.75 nm (see equation (1) in Methods in ESI†), suggesting that the microgel meshes would be permeable for small molecules of few angstroms in size without any tortuous restrictions (Fig. 2d).

Further, CD spectroscopy analysis provided conformational information of the differences in the secondary and tertiary structure of polypeptide chains in fabricating PoPM (Fig. 2e). The far-UV spectra of PoP exhibited three rotatory peaks (two negative peaks of similar magnitude at 210 and 219 nm and one positive peak at 193 nm), indicating a typical α-helical structure. This spectrum was similar to one obtained for patatin, a major component with ~40% of soluble PoP.^38^ However, a characteristic β-sheet fingerprint was observed for PoPM with one negative and one positive peak at 215 and 192 nm, respectively.^39^ As potato proteins unfold between 55 and 75 °C,^40^ the gelation temperature of 80 °C used in this study might have opened up the PoP chains and formed new bonds among the polypeptide moieties in the PoPM, which resembled a more structured β-sheet-like geometry, such features have also been observed in heat-induce potato protein-based nanofibrils.^35^ Similar observations were recorded for polyalanine-terminated α-methallyl poly(ethyleneglycol) ether (HPEG-Ala_*x*_) polymer, which underwent a conformational transition from α-helix to β-sheet structure on increasing temperature from 25 to 45 °C.^41^

The near-UV CD spectra for PoP and PoPM (Fig. S2, ESI†) demonstrate the interactions of aromatic amino acids with their surrounding environments, such as side chains, peptide bonds, *etc.* The spectrum of PoP shows three distinct regions – a positive peak around 292 nm, a negative peak around 283 nm and a second positive peak at 258 nm with a fine structure between 255 and 270 nm. This was attributed to the presence of tryptophan (trp), Tyrosine (tyr) and phenylalanine (Phe), respectively.^38^ The reversal of both positive peaks at 292 and 258 nm with reduced intensity was observed for PoPM, indicating the decreasing degree of tertiary interactions. Furthermore, the intensity of the Tyr peak was enhanced, implying a significant change surrounding the aromatic moieties in PoPM. This difference in tertiary structure was the result of the unfolding of the PoP during gelation, followed by providing a new environment of aromatic moieties in the PoPM. All the aforementioned results provide evidence of the structural difference in PoP *vs.* PoPM. It was therefore important to understand whether such structural differences would be reflected in their interfacial performance at the *n*-tetradecane–water interface.

The dynamic IFT curves for all the studied systems followed a similar pattern – IFT firstly decreased rapidly, and then declined gradually and finally, reached a plateau. It is observed that IFT at the *n*-tetradecane–water interface significantly reduced in the presence of 0.1 or 0.5 wt% PoP or PoPM (Fig. 2f). This suggests that both PoP and PoPM particles were interfacially active and underwent spontaneous adsorption. However, the initial and equilibrium IFT, which provides information about the rate of interfacial adsorption and packing of emulsifier at interface, respectively, were found to be different for the PoP *vs.* PoPM.^42^ For instance, at 0.1 wt% protein concentration, the initial IFT of PoP (31 mN m^−1^) was lower than that of PoPM (38 mN m^−1^), indicating that PoP exhibited a higher rate of adsorption to the *n*-tetradecane–water interface. This could be ascribed to the larger size (*d*_H_) and, consequently, a lower diffusion coefficient of PoPM such that they underwent a slow bulk-to-interface diffusion as compared to PoP (Fig. 2b and c).

### 3.2 Characteristics of E7 LC-in-water emulsions-stabilized with PoP and PoPM

**Fig. 3:**
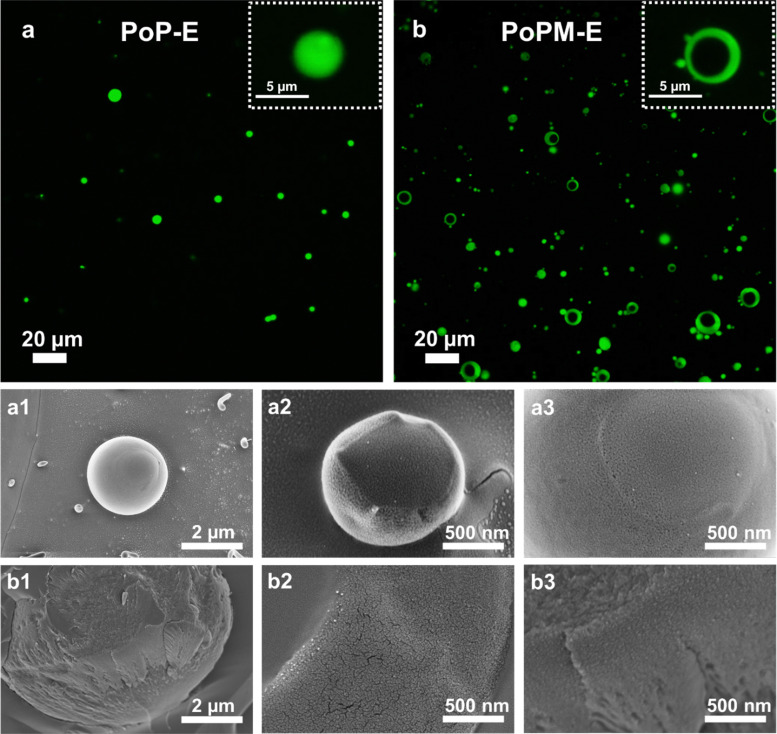
Confocal laser scanning micrographs with the zoomed images in the insets (a and b) and cryo-SEM at different magnifications (a1–a3 and b1–b3) images of E7 droplets stabilized by 0.5 wt% PoP (a and a1–a3) or PoPM (0.5 wt% potato protein concentration) (b and b1–b3), respectively.

The emulsification of E7 LC with bulk water in the presence of PoP or PoPM using a homogenization technique resulted in the formation of protein or microgel-coated emulsions. The location of PoP or PoPM particles in the prepared emulsions was determined with the help of CLSM using fast green fluorescently labelled proteins. Fig. 3a and b show the confocal image of E7-in-water emulsions in the presence of PoP and PoPM (containing 0.5 wt% protein), respectively. For both systems, E7 droplets were coated by PoP or PoPM showing the fluorescent green labelling in the confocal micrographs. In the case of PoPM (Fig. 3b), the interfacial layer was clearly visible owing to the bigger size in the range of hundreds of nanometers of PoPM (Fig. 2b and c), showing a Pickering-like stabilization. A clear-ring like interfacial layer was not visible in case of nanometric-sized PoP (Fig. 3a), as the size range is difficult to probe with confocal imaging.

Cryo-SEM imaging of these LC droplets provided further insights into the morphological features of PoP (Fig. 3a1–a3) and PoPM (Fig. 3b1–b3) at the LC–water interface. The cryo-SEM images at different magnifications show that both PoP and PoPM formed a continuous and highly inter-penetrated interfacial layer. However, a difference in morphology between PoP and PoPM at the interface was observed. In the case of PoP-coated LC droplets, a cushion with nearly distinguishable individual protein polymer (Fig. 3a3) was observed, whilst, PoPM coating had more cracks (Fig. 3b3). This could be the result of the deformation and flattening of PoPM particles while adsorbing at the interface or just the random close packing of microgels resulting in interfacial pores. In summary, both PoP and PoPM formed a compact network structure after reaching the LC–water interface, even if the stabilization mechanism involved might be different.

The E7 LC droplets coated with PoP or PoPM were monitored for the evolution of their mean size (*D*_[3,2]_ and *D*_[4,3]_), PDI and *ζ*-potential as a function of the concentration of the emulsifier as well as storage time. A series of PoP- or PoPM-stabilized E7 emulsions was prepared by varying the emulsifier concentration ranging from 0.01 to 5.0 wt%.

**Fig. 4:**
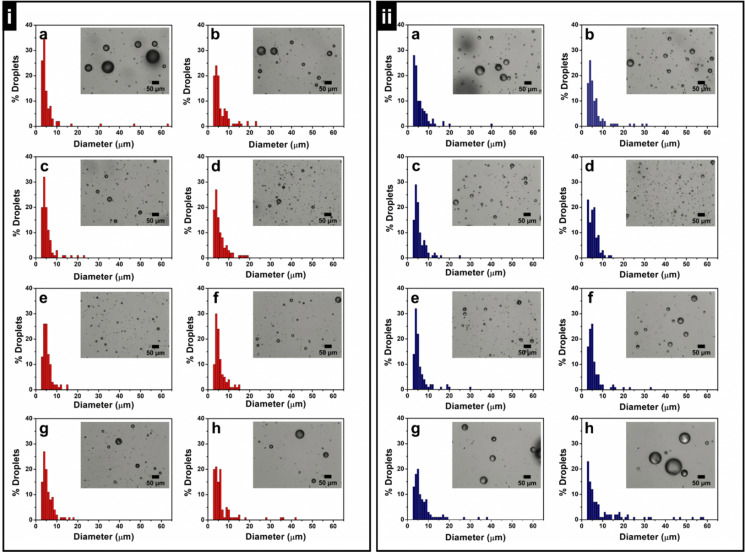
Histogram and corresponding bright field optical images of freshly prepared PoP (i) and PoPM (ii)-stabilized E7 LC-in-water emulsions with a concentration of PoP and PoPM (potato protein concentration) kept at 0.01 wt% (a), 0.05 wt% (b), 0.1 wt% (c), 0.5 wt% (d), 1.0 wt% (e), 2.0 wt% (f), 3.0 wt% (g), and 5.0 wt% (h), respectively.

Fig. 4 displays the size distribution histograms obtained by analyzing the bright field images of the PoP or PoPM-coated LC droplets. The corresponding values of *D*_[3,2]_, *D*_[4,3]_ and PDI are calculated from equations (2)–(4) (Method section in the ESI†), and *ζ*-potentials obtained from electrokinetic potential are listed in Table S1 (ESI†).

In most cases, the size of PoP-stabilized Pickering LC droplets (Fig. 4ia–h) was found to be smaller than that of PoPM-stabilized LC droplets (Fig. 4iia–h). This was associated with the larger size of PoPM as compared to the PoP coating the droplets (Fig. 2b and c). The size (*D*_[3,2]_ and *D*_[4,3]_) and PDI value of PoP- or PoPM-stabilized LC droplets initially decreased with increasing the concentration of PoP or PoPM. After a threshold concentration (*i.e.*, 1.0 wt% for PoP and 0.5 wt% for PoPM), both the E7 droplet size and PDI increased on a further increase of emulsifier concentration.

**Fig. 5:**
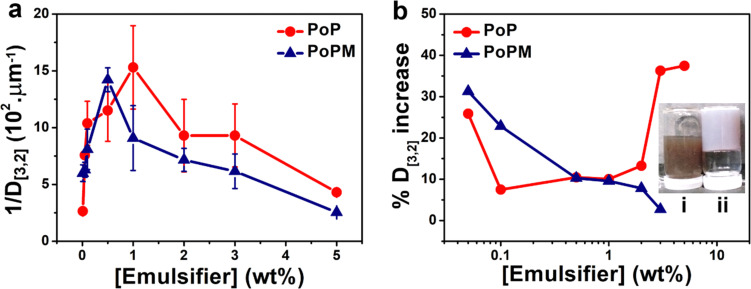
Evolution of the inverse of average drop diameter (1/*D*_[3,2]_) as a function of the initial emulsifier concentrations (a) showing the emulsifier-rich regime for PoP starting at 1.0 wt% whilst for PoPM starting at 0.5 wt% and increase in average droplet size (*D*_[3,2]_) over storage for 7 days (b) for PoP- and PoPM-stabilized emulsions as a function of emulsifier concentrations. The inset in (b) shows the vials containing 5.0 wt% PoP-stabilized emulsion (i) and 5.0 wt% PoPM-stabilized emulsion (ii) on day 7 of storage, later showing gelation. The data represents an average of at least three independent experiments on triplicate samples (*n* = 3 × 3). Error bars represent standard deviations.

In order to obtain these emulsifier-poor and -rich regimes in the prepared emulsions, the inverse of the average drop diameter (1/*D*_[3,2]_) was plotted against the initial concentration of the emulsifier (PoP or PoPM).^43^ The results presented in Fig. 5a reveal that both PoP- and PoPM-stabilized E7 droplets were present as the emulsifier-poor systems up to their respective threshold emulsifier concentration, after which they became emulsifier-rich emulsions. Thus, the initial decrease in size of LC droplets was controlled by limited coalescence, and at a threshold emulsifier concentration, the smaller droplets with large interfacial area became kinetically stable. In the emulsifier-rich regime, the droplet size was independent of emulsifier concentration. The large droplet size in the emulsifier-rich regime was related to the aggregate formation in the continuous phase.^44^ With an excess of unadsorbed microgels, the degree of microgel-microgel aggregation enhanced in the continuous phase. This led to the limited availability of microgel for adsorption at the interface, similar to a depletion-type mechanism, finally resulting in the formation of larger droplets (see Fig. 4iih).

We examined the adsorption efficiency, *α*, and effective adsorption density, *Γ*_abs_, of PoP and PoPM at the LC–water interface (see equations (5) and (6) in the Method section in ESI†). At 0.5 wt% protein concentration, the adsorption efficiency for PoP (32.6%) was found to be higher than that for PoPM (20.5%). The presence of excess emulsifier in emulsifier-poor regime (limited coalescence) for PoPM is also supported by a previous work with whey protein microgels (WPM)-stabilized oil-in-water emulsions which existed in emulsifier-poor regime with the presence of excess unadsorbed particles.^43^ The number of monolayers of PoPM at the LC–water interface can be estimated on comparison of *Γ*_abs_ with *Γ*_90%_, *i.e.*, the adsorption density of ideal PoPM monolayer at interface forming a hexagonal close packed array while covering 90% of the total interfacial area.^13,43^ Here, *Γ*_90%_ was calculated to be 9.4 mg m^−2^ using *d*_H_ of 105 nm for PoPM. A ratio *Γ*_abs_/*Γ*_90%_ ≫ 1 implies that PoPM formed a multilayer coating even at 0.5 wt% with nearly 46× monolayer at the interface. However, this calculation should be taken with caution as the microgels were not perfectly monodisperse and could have been aggregated with other microgels during the adsorption phenomena.

The *ζ*-potential values of PoP- and PoPM-coated LC droplets were higher than that of bare droplets (−17.6 ± 1.8 mV) (Table S1, ESI†), confirming the adsorption of PoP and PoPM at the LC–water interface corroborating the micrographs shown in Fig. 3. Interestingly, the *ζ*-potential values of PoP- and PoPM-coated LC droplets were greater than those of aqueous dispersion of PoP and PoPM, which further highlights that the emulsifiers were more concentrated at the interface as compared to the bulk phase. As the emulsifier concentration increased, the absolute *ζ*-potential values of the droplets increased, suggesting further adsorption of PoP or PoPM until a maxima was achieved (2.0 wt% for PoP and 1.0 wt% for PoPM). On further increase in emulsifier concentration, the absolute *ζ*-potential of droplets decreased, similar to the evolution observed in mean size values (Table S1, ESI†). These findings further support our hypothesis that the emulsifier adsorption diminished in the emulsifier-rich regime. Such emulsifier-regime detections are important to understand whether such regimes influence analyte detection, which is discussed later.

The size (*D*_[3,2]_ and *D*_[4,3]_) and PDI values of these emulsions were compared over a week of storage (Table S1 and see Fig. S3, ESI,† for the visual images of the emulsions stored over 4 weeks). Except for the 0.01 wt% emulsifier concentration, all the prepared emulsions exhibited excellent stability with a minimal change in their characteristic parameters (*D*_[3,2]_, *D*_[4,3]_, and PDI) after a period of one week. It is noteworthy that the stability of both PoP- and PoPM-stabilized LC droplets outperformed the bare as well as phospholipid-stabilized emulsions (Fig. 1). The stability of the E7 emulsions stabilized by PoP was ascribed to the electrostatic repulsions between the negatively charged emulsifier-coated LC droplets. Strikingly, PoPM-coated emulsions remained stable with a turbid milky appearance even over a storage of one month with no evidence of phase separation, giving an indication of Pickering-like stabilization.

To determine the effect of nature and concentration of emulsifier on the kinetic stability of the emulsions, the percentage increase in the size of the droplets (% *D*_[3,2]_ increase over a period of one week) was plotted as a function of emulsifier concentration (Fig. 5b). The lower the value of % *D*_[3,2]_ increase over time, the higher is the emulsion stability. For PoP-stabilized emulsions, the stability firstly increased with increasing emulsifier concentration from 0.05 to 0.1 wt%. The increased stability might be caused by the coverage of the interface by a dense layer of PoP molecules, as observed in Fig. 3a1–a3. Stability was maintained up to 1.0 wt% emulsifier concentration, which is shown by the non-significant change in *D*_[3,2]_ (*p* > 0.05) (Table S1, ESI†), after which the emulsions stability decreased with a marked increase in *D*_[3,2]_ (*p* > 0.05) (Table S1, ESI†).

In contrast, the stability of PoPM-coated Pickering LC emulsions remained fairly constant in the emulsifier-rich regime *i.e.*, at and above the threshold concentration of 0.5 wt% with a non-significant increase of *D*_[3,2]_ over time (*p* > 0.05) (Fig. 5b). All the emulsions maintained their liquid-like behavior, except the 5.0 wt% PoPM-stabilized Pickering LC emulsion system, which transformed into ‘emulsion-gel’^45^ after storage for a week (Fig. 5b). Such gelation might be attributed to the aggregation behavior of microgels, as discussed above, not only between the microgels in the continuous phase but also between microgels attached to two different droplets forming a network.^21,46^ Overall, the experimental evidences support that the Pickering stabilization by the soft PoPM provided better control over the emulsion stability as compared to those stabilized by the PoP polymer. Here, a combination of electrostatic and steric stabilization appeared to have contributed to the ultra-stability of the PoPM-stabilized LC droplets, where both *true* interfacial Pickering-like stabilization^20^ of the LC droplets by PoPM, as well as network stabilization in the bulk phase, might have contributed to the exceptional stability. Such Pickering LC droplets coated with microgels with the stability of 2 weeks has been shown in our previous work^13^ but this work shows the longer-term stability of over 4 weeks using plant-based sustainable microgels.

To evaluate whether or not the ultra-stable E7 droplets coated by PoPM still enabled the transport of aqueous analytes to the LC–water interface to induce a configurational transition within the droplets as compared to the un-microgelled PoP counterparts, the dynamic response of PoP- or PoPM-stabilized LC-in-water droplets to a model analyte (SDS) was studied. For this purpose, 0.5 (emulsifier-poor) and 3.0 wt% (emulsifier-rich) emulsifier concentrations were selected based on Fig. 5a. Also, at these concentrations, the sizes of the LC droplets were similar irrespective of the presence of PoP or PoPM (*p* > 0.05) (see Table S1, ESI†).

### 3.3 Analyte-induced ordering transition in PoP- and PoPM-coated E7 droplets

**Fig. 6:**
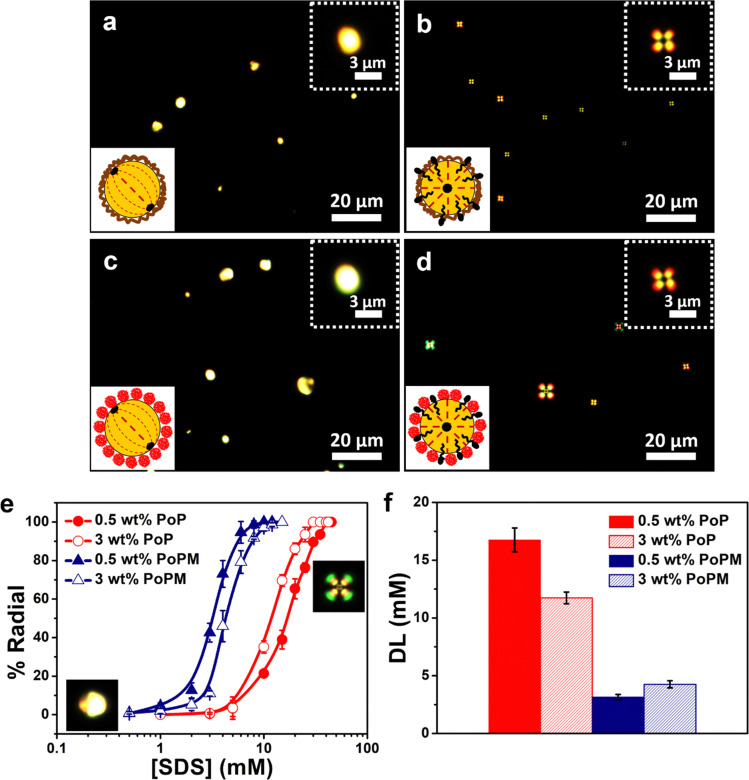
Polarized light microscopy images of the 0.5 wt% PoP- (a and b) and PoPM- (0.5 wt% potato protein concentration) (c and d) stabilized E7 droplets dispersed in (a and c) SDS-free aqueous medium with bipolar signature or (b and d) in 10 mM SDS solution with radial signature. The insets represent the schematic illustration of the PoP- and PoPM-stabilized LC droplets with respective internal configurations. Dose-response curves (e) of PoP- and PoPM-stabilized LC droplets (0.5 and 3.0 wt% potato protein concentration) for SDS measured at a fixed emulsion volume of 300 μL and detection limit (DL) (f) of PoP- and PoPM-coated LC droplets for SDS in emulsifier poor (0.5 wt%) and emulsifier rich (3.0 wt%) regimes.

In order to investigate the internal ordering of nematic LC within the droplets, the PoP- and PoPM-coated E7 droplets were observed using a cross-polarized optical microscope. As shown in Fig. 6a and c, the adsorption of PoP and PoPM did not change the internal ordering of E7 droplets, unlike the ones in the control droplets *i.e.*, the ones stabilized by phospholipids (see previous discussion for Fig. 2f). Both PoP- and PoPM-coated E7 droplets maintained their bipolar director configuration (planar surface anchoring), *i.e.*, the LC molecules were oriented parallel to the LC–water interface with two diametrically opposite surface point defects at the poles of the droplet. Similar observations were reported for chitosan^47^- and WPM^13^-coated LC droplets. In the presence of SDS, both 0.5 wt% PoP- and PoPM-stabilized E7 droplets underwent a bipolar-to-radial configuration transition (Fig. 6b and d). For the radial director configuration (with homeotropic surface anchoring), the LC molecules were oriented perpendicular to the interface with a single point defect at the center of the droplet. These droplets exhibited a characteristic ‘cross-like’ signature/pattern under the crossed polarizer. It appears that SDS molecules were able to diffuse through the interfacial layer for both PoP or PoPM and induce a parallel-to-perpendicular ordering transition of LC molecules within the droplets, resulting in a radial configuration.^13,14^ For the PoPM, the SDS could have diffused either through the interfacial holes or through the meshes as the microgels were sponge-like particles with nearly three times larger mesh size (Fig. 2d) to allow easy diffusion of the SDS molecules (radius of gyration ∼16 Å for SDS micelles)^48^ without offering any physical barrier. The competitive adsorption of SDS at the PoP- and PoPM-covered interfaces was further confirmed by the *ζ*-potential measurements (Table S2, ESI†). The absolute *ζ*-potential values of PoP- and PoPM-stabilized emulsions in both the emulsifier-poor and -rich regimes increased significantly (*p* < 0.05) in the presence of 5 mM SDS. Thus, these results highlight that both PoP- and PoPM-stabilized LC emulsions have the potential to act as LC-based optical sensors.

To have better insight into the detection limit (DL) of analytes by PoP- and PoPM-stabilized droplets, the dynamic range of LC response to SDS was obtained by measuring the number of droplets undergoing a bipolar-to-radial transition as a function of SDS concentration. Here, the emulsions were exposed to SDS solutions at a fixed emulsion volume of 300 μL. As shown in Fig. 6e, the S-shaped dose-response curves were obtained for all the studied emulsions as the percentage of bipolar-to-radial transition was plotted against SDS concentration. Interestingly, PoPM-stabilized Pickering emulsions provided a more sensitive response to SDS with a lower DL in comparison to PoP-stabilized emulsions. The DL values, *i.e.*, the SDS concentration at which 50% transition occurred, were found to be 16.8 and 11.7 mM for 0.5 and 3.0 wt% of PoP-stabilized emulsions, respectively. Whereas, the lower values of DL were obtained for PoPM-stabilized emulsions: 3.2 and 4.3 mM for 0.5 and 3.0 wt% of initial emulsifier concentrations, respectively (Fig. 6f).

The higher DL of PoP-stabilized LC emulsions for SDS could be explained by the strong interaction between PoP (protein) and SDS (anionic surfactant). Depending on the concentration of SDS, specific binding and cooperative non-specific binding interactions at low and high SDS concentrations, respectively, occurred, which might have resulted in lesser quantities of SDS being available to LC molecules for the transition.^49^

Furthermore, there is also a possibility that the SDS molecules underwent competitive adsorption at the LC–water interface by removing reversibly-adsorbed PoP from the interface. However, the protein-SDS complexes are often found to desorb less from the surface than protein or SDS alone^50^ and can be energetically more favorable than the SDS micellization.^51,52^ This implies that for the same protein concentration, a large amount of SDS is required to either diffuse through the PoP layer or to displace the pre-adsorbed PoP molecules from the interface.

On the other hand, such interactions were relatively limited in the case of microgels due to their compact structure, in which the hydrophobic moieties were most likely buried within the 3D cross-linked network by unfolding and refolding process during microgel fabrication. Further, the competitive adsorption of SDS was not occurring as the microgels were irreversibly adsorbed at the interface. The SDS molecules gained access to the LC droplets mainly by passing through the interfacial pores between the adsorbed PoPM particles at the LC–water interface or the spongy meshes, as reported earlier.^13^ The surface-active SDS molecules, once at the interface, interacted with LC and induced a bipolar-to-radial transition within the droplets.

An interesting feature between the dose-response curves of PoP- and PoPM-stabilized emulsions was observed while moving from emulsifier-poor to -rich regimes. For PoP-stabilized emulsions, the DL was shifted to a lower SDS concentration with increasing initial emulsifier concentration from 0.5 (emulsifier-poor) to 3.0 wt% (emulsifier-rich). Whereas, an opposite trend was observed for PoPM-stabilized emulsions, *i.e.*, the DL became higher in the emulsifier-rich regime (Fig. 6f). For the microgels, it might be attributed to the network stabilization interaction at such high concentration, where SDS might have been bridged between microgels in the continuous phase being less available to the encapsulated LC molecules. Such behavior of formation of percolating networks by microgels bridging other particles or surfactant molecules at higher concentrations of microgels has been reported in the literature previously.^53,54^

It is noteworthy that the DL of the plant-based microgels was slightly higher than the animal-based (whey protein) microgel- or *p*NIPAM microgel-stabilized droplets reported previously.^13,14^ For instance, the DL was 0.85 mM for the WPM and <2.5 mM for the *p*NIPAM microgel-stabilized LC droplets. However, such differences in DL are more likely to be associated with the type of LC molecules used, *i.e.*, 5CB used in previous studies, as opposed to E7 used in the current study. For comparative reasons, PoPM-stabilized droplets were produced using 5CB (data not shown), and the DL was 1.8–2.3 mM SDS for PoPM concentration ranging from 0.005 to 0.1 wt%. Therefore, the use of plant-based microgels did not influence the DL. Thus, by comparing the results of emulsion stability and response behavior between PoP- and PoPM-stabilized E7 droplets, we conclude that the PoPM can not only act as a better stabilizer (Pickering) for E7-in-water emulsions than PoP, offering excellent longer-term stability but also a lower DL for small molecular analytes similar to those of synthetic microgels.

Further, one might argue that the dynamics of the SDS-induced bipolar-to-radial transition and the effect of environmental conditions, such as pH, ionic strength, *etc.*, on the response study is not reported here. Being a statistical method for response study (% Radial), a complete transition was obtained within 30 min of incubation of SDS with coated-LC emulsions with no additional change in percentage transition with the passage of time. However, Shechter *et al.*^55^ have reported the dynamics for SDS-induced ordering transition in the bare LC droplets where the direct observation of single LC droplets held with tweezers showed that the optimum time taken for bipolar-to-radial transition was ∼11.5 min and was a function of the concentration of SDS and size of LC droplets. It will be interesting to study the dependence of transition dynamics of PoP and PoPM-coated LC droplets on various factors, such as concentration of SDS, size of droplets, *etc.*, which is beyond the scope of this study. Furthermore, there are various reports showing the effect of environmental conditions, such as ionic strength, pH, *etc.*, on the emulsification properties of the PoP and PoPM and stability of resultant emulsions, and on the response behavior of LC droplets. For instance, it has been reported that PoPM exhibits poor emulsifying property when the pH of the media is near their pI (4.5–5.0) while the PoPM-stabilized oil-in-water emulsions were found to be highly stable when the pH of the media is away from the pI.^27^ Another report mentioned that the presence of salt enhanced the surface activity of PoP, and consequently, foamability and foam stability were altered.^56^ Also, the LC droplets were found to be environment-sensitive, where a decrease in ionic strength and/or pH of the media caused a decrease in the percentage of LC droplets undergoing bipolar-to-radial transition, whilst the transition could only be obtained under specific pH and ionic strength conditions.^6^ From this, the environment sensitivity of PoP, PoPM and LC which may affect the observed stability and response of the PoP- and PoPM-coated LC-in-water emulsions needs further investigation.

**Fig. 7:**
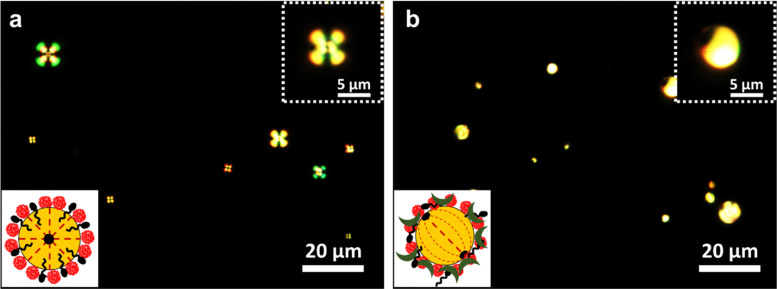
Polarized light microscopy images of the SDS/PoPM complex-stabilized Pickering LC droplets (PoPM containing 0.5 wt% potato protein and 8 mM SDS) dispersed in NaCh (a classic bile acid salt)-free aqueous medium with radial signature (a) and 20 mM NaCh with bipolar signature (b). The insets on the bottom-left side represent the schematic illustration of the PoPM-stabilized Pickering LC droplets with respective internal droplet configurations. The insets on the top-right side represent the higher magnification images of the LC droplets with respective optical signatures.

The potential of PoPM-stabilized Pickering LC emulsions was further evaluated for the detection of biological analytes. For this purpose, NaCh, sodium salt of cholic acid, *i.e.*, a primary BA, which is an important biomarker for the early diagnosis of LDs, was selected as a model biological analyte. As reported earlier,^7,47^ the general procedure for the detection of BA using LC droplets is a two-step process. Firstly, the bipolar LC droplets are exposed to surfactants (*e.g.*, SDS), triggering a radial configuration. Subsequently, the BAs are added to the aqueous solution containing surfactant-adsorbed radial droplets. The addition of BAs brings the radial droplets back to a bipolar configuration. Fig. 7 shows that the SDS/PoPM complex-coated E7 droplets (containing 0.5 wt% PoPM and 8 mM SDS) underwent a reverse transition from radial-to-bipolar in the presence of NaCh. At 20 mM NaCh, 84.3% of bipolar droplets were obtained. This reverse transition was caused by the competitive adsorption of NaCh at the LC–water interface, where the pre-adsorbed SDS molecules were removed either partially or completely from the interface. The adsorption of NaCh at the SDS-laden LC–water interface was confirmed further by the *ζ*-potential measurements. The *ζ*-potential of SDS/PoPM-coated Pickering emulsions (0.5 wt% PoPM and 5 mM SDS) reduced from −75.2 to −52.7 mV in the presence of 20 mM NaCh (*p* < 0.05) (Tables S2 and S3, ESI†). This suggests that the plant-based microgel-stabilized Pickering LC emulsions can be used as an assembly for the detection of both chemical and biological analytes in an aqueous medium. However, the polydispersity of these emulsions may influence the reliability of the results for quantitative estimation of the aqueous analytes. In order to present the developed assembly as a reliable quantitative method, an attempt was made to prepare monodisperse Pickering LC droplets *via* a microfluidic device.

### 3.4 Characterization and analyte response of pickering E7 droplets prepared by a microfluidic device

**Fig. 8:**
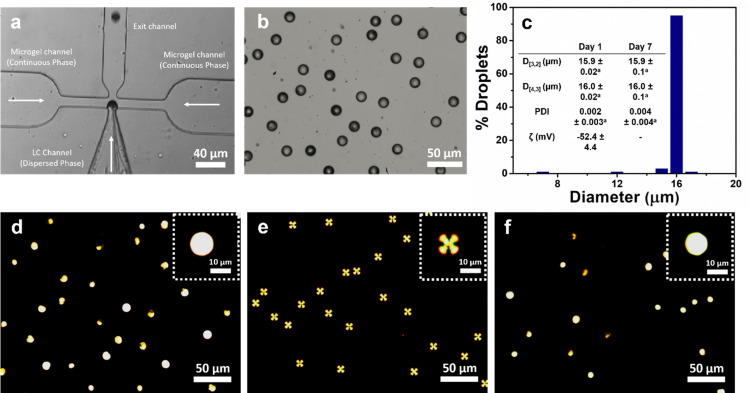
Optical image of a microfluidic device in use with a high-speed camera (a) showing the formation of a PoPM-stabilized LC droplet. The LC (E7) is injected from the middle channel, and the PoPM aqueous solution (1.0 wt% potato protein concentration in the microgel) is injected from two side channels. The bright field image of the obtained PoPM-coated E7 droplets (b) using the microfluidic device and corresponding droplet size distribution (c) with inset table showing the size (*D*_[3,2]_ and *D*_[4,3]_), PDI and *ζ*-potential values of freshly prepared Pickering E7 droplets on day 1 and after storage for a week. Samples in the same row with the same superscripts do not differ significantly (*p* > 0.05) according to Tukey's test (c). Polarized optical microscopy image of the PoPM-coated E7 droplets obtained *via* microfluidics (d), SDS/PoPM complex-coated E7 droplets in the presence of 10 mM SDS with radial signature (e) and NaCh/SDS/PoPM complex-coated E7 droplets in the presence of 20 mM NaCh and 10 mM SDS with bipolar signature (f).

Fig. 8a and b show the experimental setup of flow focus droplet microfluidic device for the preparation of Pickering emulsions and the bright field image of the resulting 1.0 wt% PoPM-coated E7 droplets, respectively. The *D*_[3,2]_, *D*_[4,3]_ and *ζ*-potential values of the freshly prepared Pickering E7 droplets were found to be 15.9 μm, 16 μm, and −52.4 mV, respectively (Fig. 8c). The *ζ*-potential values of these microfluidic-fabricated LC droplets were similar to those produced by the homogenization method (*p* > 0.05) (Table S1, ESI†), suggesting that the surface coverage was similar irrespective of the method of preparation. As expected, the emulsions exhibited an excellent monodispersity with a very low PDI of 0.002. This was further confirmed by a narrow size distribution histogram (Fig. 8c).

The *D*_[3,2]_ and *D*_[4,3]_ values remained unchanged over a period of 1 week, indicating that the droplets were stable. Similar to the previous observation, PoPM-coating within the microfluidic device did not alter the bipolar configuration of E7 droplets (Fig. 8d). Most importantly, the Pickering LC droplets that were obtained *via* microfluidics exhibited a similar response to the chemical and biological analytes as observed in case of the emulsions prepared by homogenization method. The microfluidically obtained Pickering E7 droplets underwent a bipolar-to-radial transition upon exposure to 10 mM SDS (Fig. 8e) followed by a reverse transition from radial-to-bipolar in the presence of 20 mM NaCh (Fig. 8f). Therefore, we have demonstrated the evidence to design monodisperse Pickering emulsions using plant-based microgels that can be used for detecting chemical and biological analytes by virtue of their analyte-induced optical configurational transition.

## 4. Conclusions

In summary, our findings demonstrate that the PoP-based materials provide sustainable, non-toxic and biodegradable emulsifiers for LC droplet-based optical sensors as an alternative to the known synthetic and animal-based emulsifying materials. Both PoP polymer and PoPM act as effective emulsifiers where they adsorb at the LC-aqueous interface and provide electrostatic and steric stabilization to the LC droplets. These resultant coated-LC emulsions exhibited narrow size distribution and higher shelf life than the bare and phospholipid-coated LC emulsions. Furthermore, Pickering stabilization in the case of PoPM provides better stability to the prepared PoPM-coated LC-in-water emulsions offering months of kinetic stability unachieved by any other stabilization technique to date. The penetrable interfacial coating of PoP as well as the spongy-mesh like architecture of the PoPM allows the interaction of SDS with the LC droplets and results in LC droplet configuration transition-based optical signal. Further, the Pickering LC droplets with improved stability and lower DLs respond to biological analytes, which can serve as important biomarkers for early diagnosis. The generation of ultra-stable, monodisperse Pickering LC droplets using microfluidics with the same response to chemical and biological analytes eliminated the undesirable effects of droplet polydispersity on the response of these highly sensitive sensors. The ongoing studies involve the investigation of the effect of environmental conditions on the stability, response and configuration transition dynamics of the PoP- and PoPM-stabilized Pickering LC emulsions. Overall this study, followed by ongoing series of optimization, will pave the way forward toward the application of the Pickering emulsions as a cheap sensor for sensitive and selective detection of BAs that can be used in clinical settings.

## Data availability

The data presented in this article is openly available from the University of Leeds Data Repository: https://doi.org/10.5518/1330.

## Conflicts of interest

The authors declare that they have no known competing financial interests or personal relationships that could have appeared to influence the work reported in this paper.

## Supplementary Material

TC-011-D3TC00598D-s001
