# Supplementary material for: Ultra-stable liquid crystal droplets coated by sustainable plant-based materials for optical sensing of chemical and biological analytes
Source: J Mater Chem C Mater. 2023 Apr 11;11(17):5831–45. doi: 10.1039/d3tc00598d (PMC10158717; doi:10.1039/d3tc00598d)
Supplement: TC-011-D3TC00598D-s001 [file TC-011-D3TC00598D-s001.pdf]

Electronic Supplementary Material (ESI) for Journal of Materials Chemistry C.
This journal is © The Royal Society of Chemistry 2023

Supplementary Information

Ultra-stable liquid crystal droplets coated by sustainable plant based-materials for optical sensing of chemical and biological analytes

Shikha Aery [a,b], Adele Parry [c], Andrea Araiza-Calahorra [b], Stephen D Evans [c], Helen Gleeson [c], Abhijit Dan [a,d†], Anwesha Sarkar [b*]

[a] Department of Chemistry and Centre for Advanced Studies in Chemistry, Panjab University, Chandigarh 160014, India

[b] Food Colloids and Bioprocessing Group, School of Food Science and Nutrition, University of Leeds, LS2 9JT, UK

[c] School of Physics and Astronomy, University of Leeds, LS2 9JT, UK

[d] Department of Applied Chemistry, Maulana Abul Kalam Azad University of Technology, Simhat, Haringhata, West Bengal 741249, India

Corresponding Authors

Email: abhijit@pu.ac.in (A.D.)[†]; A.Sarkar@leeds.ac.uk (A.S.)[*]

Supplementary Section 1

1. Methods

1.1 Preparation of aqueous dispersions of potato protein (PoP) and potato protein microgels (PoPM)

Aqueous solutions of potato proteins (PoP, 10.0 wt%) were prepared by dispersing potato protein isolate (PoPI) powder in Milli Q water for 2 h to ensure solubilisation. To remove any insoluble fractions, the dispersion was centrifuged at 5,000 rpm for 20 min at 25 °C. The supernatant containing the soluble fraction was carefully collected and stirred at 200 rpm for 20 min. The % solubility calculations showed that PoPI had ~ 99.89 % solubility in Milli Q water, with most of the protein solubilized in agreement with previous work.[1] The soluble supernatant fraction was used as a stock solution to create various concentrations of PoP solutions.

For fabricating the aqueous dispersion of potato protein microgels (PoPM), a slightly modified top-to-down approach described by Sarkar et al. was used.[2] For this, 10.0 wt% PoP solution was heated in temperature controlled water bath at 80 °C for 30 min to denature patatins. The resultant heat-set PoP-based hydrogel was cooled down to 25 °C and stored at 4 °C overnight. To obtain the PoPM, firstly, the hydrogel was mixed with Milli Q water (1:1 w/w) and prehomogenized using a hand blender (HB711M, Kenwood, UK) to create macrogel particles. The 50 vol% PoP macrogel particle containing 5.0 wt% PoP was degassed using a Thinky instrument (Intertronics, Thinky ARE-250, Oxfordshire, UK). Finally, the defoamed dispersion of PoP macrogel particle was homogenized by passing through a two-stage valve homogenizer (Panda Plus 2000, GEA Niro Soavi Homogeneizador, Parma, Italy) four times operating at first/second stage pressures of 200/100 bars, respectively. The resultant solution was termed as 5.0 wt% PoPM aqueous solution, i.e., microgel particles containing 5.0 wt% protein, with a volume fraction of 50 vol% PoPM, which was diluted with Milli Q water at pH

6.15 to create various concentrations of the microgels for fabricating Pickering LC emulsions. Sodium azide (0.02 wt%) was added as a preservative to both PoP (10.0 wt%) and PoPM (5.0 wt%) solutions to inhibit bacterial growth.

1.2 Characterization of PoP solution and PoPM dispersion

1.2.1 Particle size. The particle size of PoP and PoPM was obtained using dynamic light scattering (DLS). The size distribution, hydrodynamic diameter (d_{H}) and polydispersity index (PDI) of PoP and PoPM was measured using a Zetasizer Nano-ZS (Malvern Instruments Ltd., Worcestershire, UK) instrument operating at a detection angle of 173° while using 633 nm He-Ne laser light source. Firstly, both PoP and PoPM stock solutions were diluted to 0.01 wt% protein concentration using Milli Q water. Further, these solutions were analyzed at 25 °C using standard disposable DTS0012 plastic cuvettes with fixed absorbance and refractive index values of protein to 0.001 and 1.45, respectively.

1.2.2 Mesh size of PoPM. The mesh size of the PoPM particles was calculated indirectly by considering that PoPM is an average nanometric unit of the PoP-hydrogel, and no syneresis occurred during the formation of the microgels.[3] The average mesh size, ξ, is related to the storage modulus, G', obtained for the PoP hydrogel as given by the following equation:[4,5]

$$\xi^3 = \frac{K_B T}{G'} \quad (1)$$

where, K_{B} and T are Boltzmann constant and temperature, respectively.

To measure G', the thermal gelation of PoP in the rheometer was followed by a frequency sweep test of the PoP gel. For this, a modular compact rheometer, MCR 302 (AntonPaar, Graz, Austria) equipped with a cone and plate (CP 50, 50 mm diameter and cone angle 2°) geometry was used. For gelation, 10.0 wt% PoP aqueous solution was placed in the

measuring cell carefully to avoid bubbles formation. The silicone oil and adiabatic hood were used to seal the cell to prevent evaporation. Firstly, the cell temperature was increased from 25 °C to 80 °C, followed by keeping the cell at 80 °C for 10 min. After PoP gelation, the cell temperature was reduced to 25 °C before the frequency sweep test was performed with varying angular frequency, ω from 1 to 100 rad/s keeping the shear strain constant at 0.1 %. The G' and loss modulus, G" for PoP-gel were obtained where the final G' value at 100 rad/s was used to calculate mesh size.

1.2.3 Circular dichroism. To understand the heat-induced conformational change in the secondary and tertiary structure of PoP while forming PoPM, the far and near UV circular dichroism (CD) spectra of PoP solution and PoPM dispersion were recorded, respectively, using Chirascan Plus, Applied photophysics Spectropolarimeter (Leatherhead, UK). For analysis, 0.025 wt% of PoP solution and PoPM dispersion were prepared using Milli Q water as solvent. For far UV spectra, the measurements were performed using a 1 mm path length cell at 180 – 260 nm, while for near UV spectra, a 10 mm path length cell at 240 – 350 nm was used. Both analyses were carried out using quartz cuvettes and with the temperature maintained at 20 °C, 2 nm bandwidth and 1 nm step size. The obtained spectra were corrected by subtracting Milli Q water as the baseline.

1.2.4 Dynamic interfacial tension measurements. For comparison of interfacial properties of PoP and PoPM, the dynamic interfacial tension (IFT) between *n*-tetradecane and water in the presence of different concentrations of PoP and PoPM was measured using the Pendant drop method. Dataphysics OCA tensiometer (DataPhysics Instruments, Filderstadt, Germany) was used with an inbuilt experimental cell, optical and data acquisition system, and fitted syringe (Hamilton 500 μL, DS 500/GT) and dosing needle (diameter 1.65 mm). Before measurement, 0.1 and 0.5 wt% PoP solutions and PoPM dispersions were prepared using Milli Q water. The aqueous dispersions (~ 20 μL) of PoP or PoPM were extruded from a syringe at a dosing rate

of 2 μL/sec resulting in the formation of a drop at the tip of needle immersed in a cuvette containing nearly 5 mL *n*-tetradecane oil. The shape of the droplet is a result of a balance between IFT and gravity acting on the droplets. The droplet profile was imaged over time, and the contour value obtained from instrument SCA 22 software was fitted to the Young-Laplace equation resulting in dynamic IFT values. All measurements were made at 22 °C and atmospheric pressure, and over 1800 sec until a steady IFT was attained.

1.3 Preparation of LC-in-water emulsions

1.3.1 High pressure homogenization. The mixture of 20 μL E7 LC and 5 mL of an aqueous dispersion of PoP or PoPM or Milli Q water (as a control for the bare emulsion) was homogenized at 8,000 rpm for 1 min at room temperature using Ultra turrax homogenizer (IKA-Werke GmbH & Co., Staufen, Germany) with 10 mm head (S25N-10G), resulting in coated or bare E7-in-water emulsions, respectively. The concentration of PoP and PoPM in emulsions was varied from 0.01 to 5.0 wt%, for which the PoP (10.0 wt%) and PoPM (5.0 wt%) stock solutions were diluted using Milli Q water.

1.3.2 Microfluidics. Monodisperse PoPM-stabilised LC emulsions were produced using a flow-focus polydimethylsiloxane (PDMS)-based microfluidic device. Additionally, phospholipid-stabilized LC droplets using DOPC/DOPG (1:1) as control were also fabricated.[6] Liposomes were prepared from straight-chain phospholipids (1:1 DOPC:DOPG) following the protocol described by Bao et al.[7] The microfluidic device contained two inlets, one for the flow of the phospholipid liposomes (5 mg/mL) or the PoPM (1.0 wt%) onto the chip through side channels, and the other for the E7 LC through a middle channel. Flow onto the chip was controlled by two PHD ULTRA syringe pumps (Harvard Apparatus, US) with flow rates for the LC of 0.1 μL/min and 10 μL/min for the phospholipid liposomes and PoPM, respectively. The glass syringes used for both the LC and the phospholipids / PoPM had volumes of 250 μL

and 1 mL, respectively. Each syringe was fitted with a 23 gauge needle and PTFE tubing that fitted snuggly into the microfluidic device. To form droplets, the E7 LC was pinched off by the shear forces from the phospholipid liposomes and PoPM, which then adsorbed at the LC/aqueous interface to create a stable DOPC/DOPG or Pickering PoPM-coated LC droplets that travelled up the exit channel to the outlet. Droplets were collected in a 1.5 mL centrifuge tube for further study.

1.4 Characterization of E7-in-Water emulsion

1.4.1 Confocal laser scanning microscopy. The location of PoP and PoPM in the E7-in-water emulsions was determined using the Zeiss LSM880 confocal microscope (Carl Zeiss MicroImaging GmbH, Jena, Germany) operating in upright mode. For imaging, emulsions containing 0.5 wt% of PoP and 5.0 vol% PoPM (*i.e.*, containing 0.5 wt% protein) were used. The emulsions (600 µL) were placed on microscope slides and stained with 8 µL of an aqueous dispersion of Fast green (1 mg/mL). Further, 100 µL of xanthan gum (1.0 wt%) was added to the stained emulsions as a fixator to hinder the movement of E7 droplets. After mixing, the emulsions were secured with coverslips and placed on the microscope. The emulsions were excited at 633 nm at 25 °C, and emission filters were set at 660-710 nm. For imaging, an oil immersion 63× objective lens was used while maintaining 1 airy unit of pinhole diameter to filter out the scattered lights. The collected images were analyzed using the FiJi software.

1.4.2 Cryogenic-scanning electron microscopy (Cryo-SEM). Cryogenic-scanning electron microscopy (cryo-SEM) of the PoP and PoPM-stabilized E7-in-water emulsions containing 0.5 wt% protein, respectively, was conducted for morphological characterization of the adsorbed PoP and PoPM at the E7-water interface. For this, an FEI Helios G4 CX Dual Beam Field Emission Gun Scanning Electron Microscope (FEGSEM) attached with a Quorum Technologies PP3010 cryo system was used. The emulsion samples (~ 10 µL) were mounted

onto the copper rivets and attached to a cryo shuttle. The samples were then plunge-frozen into slushed nitrogen at approximately -207 °C. Once frozen, samples were drawn into a vacuum pot and transferred to the cryo-preparation chamber. The chamber had been pre-cooled to -140 °C on the stage and to -175 °C at the anti-contaminator, and pumped to 10^{-7} mbar vacuum. The frozen emulsions were fractured using a cooled knife and coated with Iridium to prevent samples charging (5 mA for 45 s). Finally, the coated samples were transferred to the SEM for imaging at -140 °C.

1.4.3 Droplet size measurement. To determine the size distribution and PDI of E7 droplets in PoP and PoPM-stabilized emulsions with variation of emulsifier concentration (0.01 – 5.0 wt%) and storage time (day 1 and 7), 8 µL of the emulsion was placed between a glass cover slip and microscope glass slide and sealed using a spacer. The emulsions were imaged using Leica DM 2700 P microscope (Wetzlar, Germany) operating in transmission mode with 50× objective and DeltaPix Invenio 3SII camera (Smørum, Denmark). 100 different bright field images of each emulsion were taken and analysed using the ImageJ software to obtain the diameter of the individual E7 droplets. The droplet size distribution and polydispersity of emulsions were reported in terms of Sauter mean diameter, $D_{[3,2]}$, and De Broucker mean diameter, $D_{[4,3]}$, and PDI, respectively, using equations 2, 3 and 4:

$$D_{[3,2]} = \frac{\sum_i N_i D_i^3}{\sum_i N_i D_i^2} \tag{2}$$

$$D_{[4,3]} = \frac{\sum_i N_i D_i^4}{\sum_i N_i D_i^3} \tag{3}$$

$$PDI = \left(\frac{\sigma}{D_{av}}\right)^2 \tag{4}$$

where, N_i is the number of droplets with diameter D_i, σ is the standard deviation in obtained diameter data, and D_{av} is the average mean droplet diameter.

1.4.4 Adsorption efficiency and density of PoP and PoPM. Interfacial adsorption of PoP and PoPM at the surface of E7-in-water emulsions was determined by measuring the concentration of unadsorbed PoP and PoPM left in the continuous aqueous phase of emulsion ($c_{unadsorbed}$) and subtracting this from the concentration PoP and PoPM originally used to prepare the corresponding emulsions ($c_{initial}$). The prepared PoP and PoPM-stabilized LC-in-water emulsions (0.5 wt% protein) were centrifuged at 2,100 rpm for 6 min at 25 °C. The supernatants were carefully collected using a syringe, and their absorbance was recorded using a UV-Vis Spectrophotometer (Multiskan FC microplate photometer, Thermo Scientific) at 595 nm using a standard Bradford assay kit. The protein concentrations in the supernatants were determined using the Lowry method with Bovine serum albumin (BSA) as a standard. The adsorption efficiency, α, defined as the percentage of emulsifier (PoP and PoPM) adsorbed to the LC droplets relative to the total added content and the effective adsorption density, Γ_{ads}, *i.e.*, the density of emulsifier at the interface, were evaluated using equation (5) and (6), respectively as reported elsewhere.[3]

$$\alpha = \frac{c_{adsorbed}}{c_{initial}} \times 100 \tag{5}$$

$$\Gamma_{ads} = \left(\frac{1 - \phi}{6\phi}\right) D_{[3,2]} c_{adsorbed} \qquad (6)$$

where, $c_{adsorbed}$ and $c_{initial}$ are the concentration of the adsorbed and initially added emulsifier (PoP and PoPM) in the emulsion, respectively with $c_{adsorbed} = c_{initial} - c_{unadsorbed}$. ϕ is the volume fraction of the LC phase, and $D_{[3,2]}$ is the Sauter mean diameter of the LC droplets.

1.5 ζ-potential. The ζ-potential of the protein, microgel, and the E7-in-water emulsions with or without analytes was evaluated using Zetasizer Nano-ZS (Malvern Instruments Ltd., Worcestershire, UK) instrument operating at a detection angle of 173° and with 633 nm He-Ne laser light source using folded capillary cell DTS 1070. For PoP and PoPM, the stock solutions were diluted to 0.01 wt% protein concentration, and for emulsions, all stabilized emulsions were diluted ten-fold while the bare emulsion was diluted five-fold prior to measurements. In the presence of analytes, the emulsions were incubated with analytes for 30 min before the ζ-potential measurement.

1.6 Statistical analyses. All measurements were performed three times on triplicate samples and reported as means and standard deviations ($n = 3 \times 3$). The statistical analyses were conducted using one-way (ANOVA), and samples were considered to be significantly different with $p < 0.05$ using the Tukey test.

Table S1. Physicochemical characteristics of E7 LC-in-water emulsions stabilized by different concentrations (0.01, 0.05, 0.1, 0.5, 1.0, 2.0, 3.0 and 5.0 wt%) of PoP or PoPM on day 1 and day 7. The values represent means ± standard deviations of at least three independent experiments on triplicate samples (n = 3 × 3). Different samples in the same row with the same superscripts do not differ significantly ($p > 0.05$) according to Tukey's test.

**[PoP] (wt%)**		**0.01**	**0.05**	**0.1**	**0.5**	**1**	**2**	**3**	**5**
**Day 1**	$D_{[3,2]}$ (μm)	38.1 ± 4.1[a]	13.2 ± 0.5[bc]	9.8 ± 1.8[c]	8.9 ± 2.1[c]	6.7 ± 1.6[c]	11.4 ± 3.9[c]	11.2 ± 3.4[c]	23.3 ± 1.6[b]
	$D_{[4,3]}$ (μm)	53.4 ± 0.6[a]	18.0 ± 2.4[c]	13.1 ± 3.3[c]	11.0 ± 2.7[c]	7.9 ± 2.3[c]	15.7 ± 7.6[c]	15.3 ± 6.1[c]	33.8 ± 1.4[b]
	PDI	1.7 ± 0.36[a]	0.5 ± 0.04[bc]	0.4 ± 0.11[bc]	0.3 ± 0.07[bc]	0.2 ± 0.07[c]	0.4 ± 0.19[bc]	0.4 ± 0.22[bc]	0.9 ± 0.08[b]
	ζ (mV)	-23.0 ± 2.4[d]	-27.1 ± 1.9[d]	-35.7 ± 3.7[c]	-45.5 ± 4.0[ab]	-48.1 ± 4.6[a]	-49.8 ± 0.9[a]	-47.8 ± 1.7[a]	-39.7 ± 1.8[bc]
**Day 7**	$D_{[3,2]}$ (μm)	Unstable emulsion (LC re-separation)	16.6 ± 11.6[ab]	10.5 ± 2.5[ab]	9.9 ± 0.4[ab]	7.4 ± 0.2[b]	12.9 ± 3.8[ab]	15.3 ± 7.9[ab]	32.0 ± 6.8[a]
	$D_{[4,3]}$ (μm)		23.5± 16.8[ab]	14.4 ± 3.6[ab]	14.3 ± 0.7[ab]	9.5 ± 0.2[b]	17.1 ± 5.5[ab]	22.8 ± 11.2[ab]	44.6 ± 6.0[a]
	PDI		0.7 ± 0.54[a]	0.4 ± 0.15[a]	0.4 ± 0.03[a]	0.2 ± 0.01[a]	0.5 ± 0.16[a]	0.6 ± 0.32[a]	1.3 ± 0.52[a]
**[PoPM] (wt%)**		**0.01**	**0.05**	**0.1**	**0.5**	**1**	**2**	**3**	**5**
**Day 1**	$D_{[3,2]}$ (μm)	16.8 ± 2.1[b]	15.9 ± 1.7[b]	12.7 ± 2.8[b]	7.1 ± 0.5[b]	11.6 ± 3.6[b]	14.1 ± 1.9[b]	16.7 ± 4.1[b]	39.4 ± 1.6[a]
	$D_{[4,3]}$ (μm)	24.9 ± 5.7[b]	22.3 ± 1.1[b]	17.2 ± 3.1[b]	7.9 ± 0.8[b]	15.7 ± 6.2[b]	20.7 ± 2.7[b]	23.1 ± 5.9[b]	53.3 ± 9.3[a]
	PDI	0.7 ± 0.01 [ab]	0.6 ± 0.07[ab]	0.5 ± 0.16[b]	0.2 ± 0.04[b]	0.4 ± 0.18[b]	0.5 ± 0.11[b]	0.6 ± 0.09[ab]	1.2 ± 0.3[a]
	ζ (mV)	-19.1 ± 1.5[d]	-29.4 ± 0.6[c]	-39.2 ± 2.0[b]	-49.9 ± 1.9[a]	-53.1 ± 1.5[a]	-50.2 ± 6.4[a]	-39.9 ± 1.2[b]	-33.1 ± 2.5[bc]

**Day 7**	$D_{[3,2]}$ (µm)	Unstable emulsion (LC re-separation)	20.9 ± 8.5[a]	15.6 ± 4.8[a]	7.8 ± 0.2[a]	12.7 ± 1.6[a]	15.2 ± 3.5[a]	17.2 ± 5.9[a]	Gelled
	$D_{[4,3]}$ (µm)		31.7 ± 14.0[a]	23.2 ± 7.7[a]	9.6 ± 0.5[a]	19.3 ± 3.2[a]	21.7 ± 5.6[a]	21.7 ± 9.5[a]	
	PDI		0.8 ± 0.4[a]	0.6 ± 0.26[a]	0.2 ± 0.01[a]	0.5 ± 0.08[a]	0.6 ± 0.14[a]	0.6 ± 0.27[a]	

Table S2. Mean ζ-potential values of 0.5 and 3.0 wt% PoP-E and PoPM-E in the presence of 5 mM SDS. Data is represented as means and standard deviation of three independent measurements (n = 3 x 3). Samples in the same row with different superscripts differ significantly (*p < 0.05*) according to Tukey's test.

**Sample**	**ζ (mV) at emulsifier concentration of**	
	**0.5 wt%**	**3 wt%**
PoP-E	-65.9 ± 2.4^{b}	-68.2 ± 3.2^{a}
PoPM-E	-75.2 ± 3.9^{a}	-64.2 ± 3.7^{b}

*Note. For bare emulsion, ζ-potential value changed from -17.6 ± 1.8 mV to -89.7 ± 1.3 mV in the presence of 5 mM SDS. Here, all emulsions were diluted to 0.05 wt% droplet concentration for measurement except bare emulsion, which was diluted to 0.01 wt% droplet concentration.

Table S3. Mean ζ-potential values of 0.5 wt% PoPM-E in the presence of NaCh (20 mM) and both SDS and NaCh. Data is represented as means and standard deviation of three independent measurements (n = 3 x 3). Samples with different superscripts differ significantly ($p < 0.05$) according to Tukey's test.

**Sample**	**ζ (mV)**
0.5 wt% PoPM-E + 20 mM NaCh	-26.3 ± 1.9[a]
0.5 wt% PoPM + 5 mM SDS + 20 mM NaCh	-52.7 ± 2.1[b]

*Note. In the first study, the emulsions were analyzed after incubation with the analytes for 30 min. For the second study with SDS and NaCh, firstly, the emulsion was incubated with SDS for 30 min., and then NaCh was added. The measurements were made after 30 min of NaCh addition. Here, all the emulsions were diluted ten folds for the measurement.

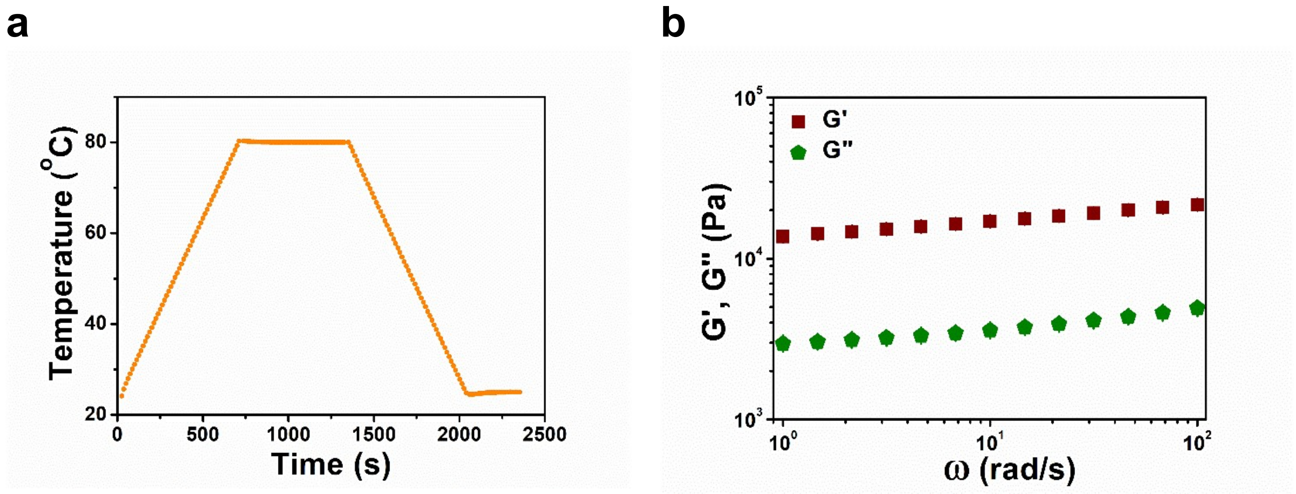

Fig. S1 Temperature sweep (a) and frequency sweep (b) to prepare 10.0 wt% heat-set PoP gel with storage modulus (*G′*) and loss modulus (*G″*) reported in b as a function of angular frequency (ω). Data represents the mean and standard deviations of three independent readings (n = 3 x 3).

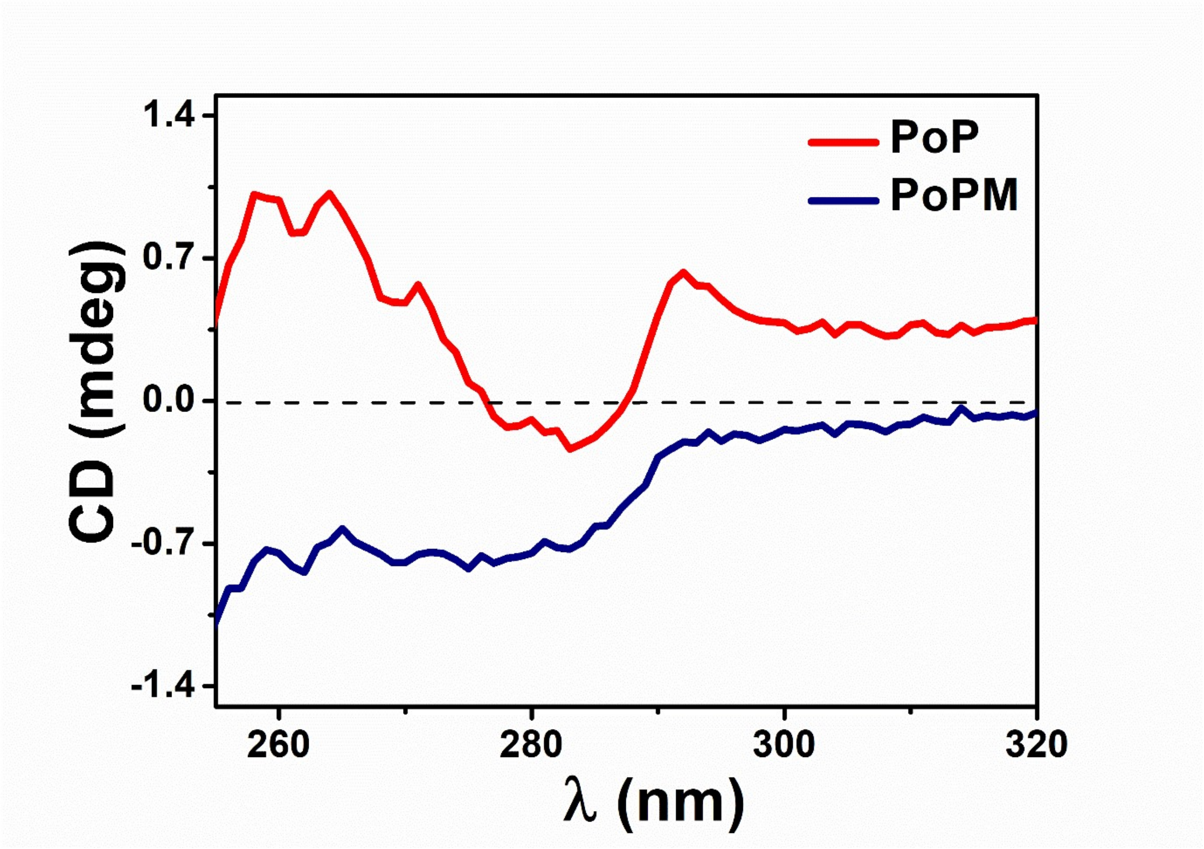

Fig. S2 The near UV CD spectrum of 0.025 wt% PoP and aqueous dispersions of PoPM, respectively.

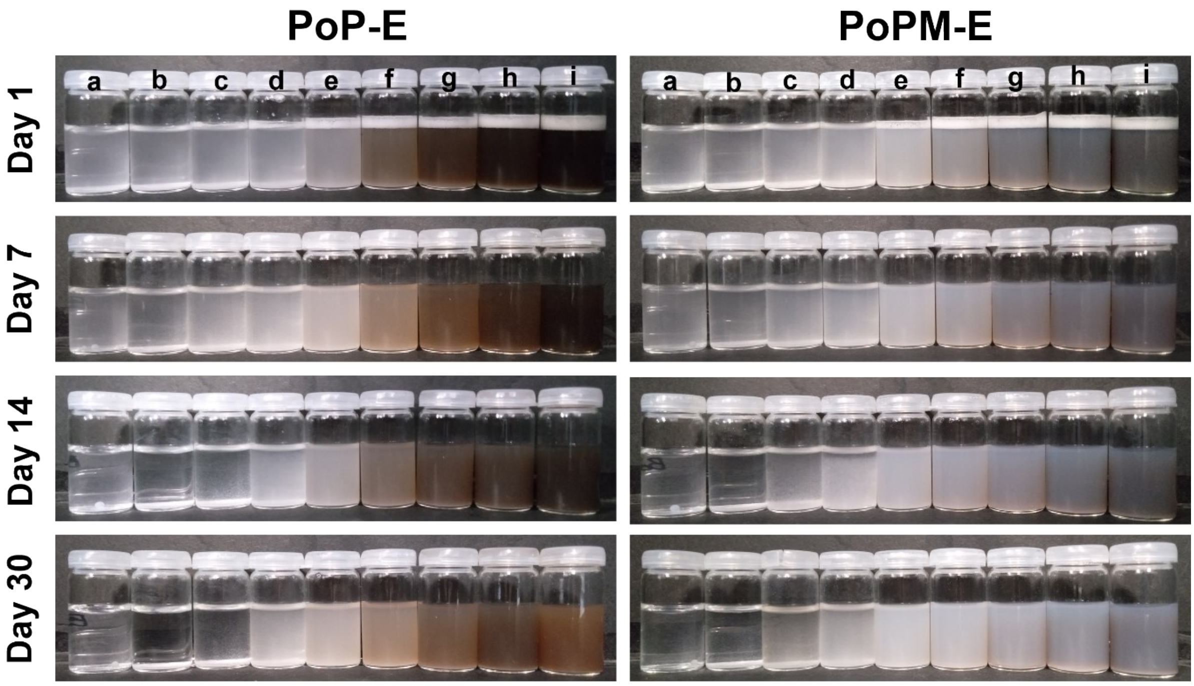

Fig. S3 Visual images of E7-in-water emulsions (bare (a) and in the presence of PoP and PoPM (b-i)) as a function of storage. The picture shows the change in the appearance of emulsions in the vial (bare emulsion) (a) and emulsions containing PoP or PoPM, (0.01 wt% emulsifier, (b)), (0.05 wt% emulsifier, (c)), (0.1 wt% emulsifier, (d)), (0.5 wt% emulsifier, (e)), (1.0 wt% emulsifier, (f)), (2.0 wt% emulsifier, (g)), (3.0 wt% emulsifier, (h)), and (5.0 wt% emulsifier, (i)) on day 1, 7, 14 and 30, respectively.

References

1. B. Kew, M. Holmes, M. Stieger and A. Sarkar, Oral tribology, adsorption and rheology of alternative food proteins, *Food Hydrocoll.*, 2021, **116**, 106636.
2. A. Sarkar, F. Kanti, A. Gulotta, B. S. Murray and S. Zhang, Aqueous lubrication, structure and rheological properties of whey protein microgel particles, *Langmuir*, 2017, **33**, 14699-14708.
3. A. Dan, S. Aery, S. Zhang, D. L. Baker, H. F. Gleeson and A. Sarkar, Protein microgel-stabilized Pickering liquid crystal emulsions undergo analyte-triggered configurational transition, *Langmuir*, 2020, **36**, 10091-10102.
4. A. Sarkar, J.-M. Juan, E. Kolodziejczyk, S. Acquistapace, L. Donato-Capel and T. J. Wooster, Impact of protein gel porosity on the digestion of lipid emulsions, *J. Agric. Food Chem.*, 2015, **63**, 8829-8837.
5. M.-C. Klak, J. Picard, S. Giraudier and V. Larreta-Garde, Mastered proteolysis of gelatin gel can control delivery kinetics of entrapped large molecules, *Soft Matter*, 2012, **8**, 4750-4755.
6. D. A. Paterson, P. Bao, R. H. Abou-Saleh, S. A. Peyman, J. C. Jones, J. A. T. Sandoe, S. D. Evans, H. F. Gleeson and R. J. Bushby, Control of director fields in phospholipid-coated liquid crystal droplets, *Langmuir*, 2020, **36**, 6436-6446.
7. P. Bao, D. A. Paterson, P. L. Harrison, K. Miller, S. Peyman, J. C. Jones, J. Sandoe, S. D. Evans, R. J. Bushby and H. F. Gleeson, Lipid coated liquid crystal droplets for the on-chip detection of antimicrobial peptides, *Lab Chip.*, 2019, **19**, 1082-1089.
